# Global hydroclimatic response to tropical volcanic eruptions over the last millennium

**DOI:** 10.1073/pnas.2019145118

**Published:** 2021-03-08

**Authors:** Ernesto Tejedor, Nathan J. Steiger, Jason E. Smerdon, Roberto Serrano-Notivoli, Mathias Vuille

**Affiliations:** ^a^Department of Atmospheric and Environmental Sciences, University at Albany, Albany, NY 12222;; ^b^Lamont-Doherty Earth Observatory, Columbia University, Palisades, NY 10964;; ^c^Institute of Earth Sciences, Hebrew University of Jerusalem, 9190401 Jerusalem, Israel;; ^d^Department of Geography, Autonomous University of Madrid, 28049 Madrid, Spain

**Keywords:** volcanic impacts, hydroclimate, Paleo data assimilation, global

## Abstract

Large tropical volcanic eruptions can affect the climate of many regions on Earth, yet it is uncertain how the largest eruptions over the past millennium may have altered Earth’s hydroclimate. Here, we analyze the global hydroclimatic response to all the tropical volcanic eruptions over the past millennium that were larger than the Mount Pinatubo eruption of 1991. Using the Paleo Hydrodynamics Data Assimilation product (PHYDA), we find that these large volcanic eruptions tended to produce dry conditions over tropical Africa, Central Asia and the Middle East and wet conditions over much of Oceania and the South American monsoon region. These anomalies are statistically significant, and they persisted for more than a decade in some regions. The persistence of the anomalies is associated with southward shifts in the Intertropical Convergence Zone and sea surface temperature changes in the Pacific and Atlantic oceans. We compare the PHYDA results with the stand-alone model response of the Community Earth System Model (CESM)-Last Millennium Ensemble. We find that the proxy-constrained PHYDA estimates are larger and more persistent than the responses simulated by CESM. Understanding which of these estimates is more realistic is critical for accurately characterizing the hydroclimate risks of future volcanic eruptions.

## Significance

Future large tropical volcanic eruptions will induce global hydroclimatic changes, superimposed on anthropogenic climate change. Understanding how volcanic eruptions affect global hydroclimate is therefore critically important. Tejedor et al. use a new paleoclimatic product, which combines information from high-resolution proxies and climate models, to estimate volcanic impacts on hydroclimate over the last millennium. They find that past eruptions caused severe drying in tropical Africa and across Central Asia and the Middle East and significantly wetter conditions over Oceania and the South American monsoon region, some of which persisted for a decade or longer. These proxy-based findings suggest that, relative to estimates from a state-of-the-art climate model, much larger and persistent hydroclimatic changes are possible across regions of important socioeconomic activity.

Explosive volcanic eruptions alter the climate by injecting large amounts of sulfur-containing gases, such as SO_2_ and H_2_S, into the stratosphere, leading to the formation of liquid sulfate aerosols (1). These aerosols scatter incoming solar radiation and absorb infrared radiation, thereby warming the stratosphere and cooling Earth’s surface because of a net decrease in the magnitude of downward shortwave radiation (2). Except for the Mount Pinatubo eruption in 1991, tropical volcanic events (TVEs) during the twentieth century were much smaller in magnitude than many earlier eruptions during the last millennium. Historical volcanic events, nevertheless, imply that large eruptions will occur again in the future and could significantly disrupt global society (3, 4) and thus potentially exacerbate long-term trends associated with anthropogenic climate change. In addition to their direct effects, volcanic eruptions have the potential to perturb large-scale ocean–atmosphere climate phenomena (5–7) that, in turn, may affect global rainfall and temperature patterns as well as cyclone activity (8). The volcanic impacts on these phenomena, such as the El Niño–Southern Oscillation (ENSO), are nevertheless the subject of persistent and unresolved debate (e.g., refs. 9, 10).

Over the instrumental period (starting around 1880 for surface temperatures and in the first half of the twentieth century for most hydroclimate records), only five tropical eruptions (Krakatau, Santa María, Agung, El Chichón, and Pinatubo) ejected climatologically important amounts of dust and sulfate aerosols into the atmosphere. Multiple studies have evaluated their impacts on the climate system, mainly focusing on associated changes in atmospheric temperatures or the major climatic modes (see ref. 7 for a recent review). Global cooling is a well-documented consequence of volcanic eruptions, with peak cold anomalies occurring between 1 to 2 y after a volcanic event. Temperature anomalies can persist for multiple years after peak cooling, illustrating that because of the inertia of the climate system, and notably the ocean, volcanic impacts on climate can persist even after the direct radiative forcing from volcanic aerosols has dissipated (7). On the other hand, studies using both models and observations for twentieth century volcanism found contrasting results on the volcanically induced global precipitation response (11, 12), in part because there are not enough observed volcanic events to make robust statistical analyses.

To overcome these shortcomings, last millennium volcanic events that are much larger than those of the twentieth century have been studied using either climate models (e.g., refs. 9, 13–16) or proxy records (e.g., refs. 17–19). Most of the work employing proxy records over the last millennium has focused on temperature responses to volcanic eruptions (e.g., refs. 18, 20, and 21), while the associated hydroclimatic impacts have received less attention and have been regionally focused (22–26). Additionally, recent proxy-based estimates of the impacts of very large volcanic eruptions have been largely limited to the boreal summer growing season (June, July, and August [JJA]) and the Northern Hemisphere (18). With the exception of proxy studies focusing on ENSO (9, 10, 27), the hydroclimate impacts of such volcanic eruptions are unknown in most of the Southern Hemisphere and much of the global tropics. Proxy and climate model comparison studies have found inconsistent hydroclimate responses over Southeast Asia (24), North America (9), and the tropical Pacific (10).

Given the uncertainties in estimated volcanic forcings, some of the above-mentioned challenges in state-of-the-art Earth System Models (ESMs), limitations in proxy sampling in space and time [including large gaps over oceans, the tropics, and the Southern Hemisphere (28)], and proxy uncertainties, proxies and models are both individually limited in their ability to advance our understanding of the hydroclimatic response to large volcanic eruptions. An improved understanding of such a response is nevertheless critical in order to better prepare society for the impacts of future large eruptions. Data assimilation products have the potential to overcome some of the abovementioned challenges by combining proxy and model information to derive spatiotemporally complete reconstructions of multiple state variables that are dynamically consistent and physically plausible. We, therefore, employ the Paleo Hydrodynamics Data Assimilation product [PHYDA (29)] (see *Materials and Methods*) to perform the first investigation of the global and seasonal impacts of large volcanic eruptions on hydroclimate using a data assimilation product. We characterize large hydroclimate impacts associated with volcanism and demonstrate that significant wet or dry anomalies can persist for a decade or more in some locations. The global and multivariate character of PHYDA allows us to interpret these changes in the context of shifts in the Intertropical Convergence Zone (ITCZ) and sea surface temperature (SST) changes in the Pacific and Atlantic oceans. These results are compared to the volcanic responses in an exclusively model-based estimate from the Community Earth System Model Last Millennium Ensemble (CESM-LME), which allows for the first global comparison of hydroclimate responses in a model and proxy-based product.

## Results

The impacts of volcanic events on global hydroclimate are determined by applying Superposed Epoch Analysis (SEA) (30) to the Palmer Drought Severity Index (PDSI, only over land) estimated in PHYDA and calculated from CESM output (see *Materials and Methods*). Additionally, we use the most up-to-date volcanic reconstruction “eVolv2k_v2” (1), which refines the dating and magnitude estimates of volcanic eruptions compared to prior volcanic reconstructions (31, 32). To perform the same analysis using CESM, we select the 13 adjacent volcanic events in the ref. 31 reconstruction because it was used to force the CESM-LME, although we also assess results using an event selection based exclusively on the ref. 31 reconstruction (*Materials and Methods* and *SI Appendix*, Table S2). We initially and explicitly compare PHYDA to the CESM-LME member 10 because it was used as the PHYDA data assimilation prior, thereby ensuring that any differences in the estimated responses are specifically the result of the proxy information added to the data assimilation product, but a comparison to all members of the LME is also explored. The SEA is based on 13 tropical (+25° N to −25° S) volcanic events greater than Pinatubo [TVEP; volcanic stratospheric sulfur injections (VSSI) > 8.78 Tg S (1)] that occurred from 1000 to 1850 CE. We exclude the first event in six “double events,” defined as eruptions that occur within 10 y of each other, to avoid potentially biasing the resulting climatic response. We perform the same analysis using all 19 eruptions (including the six double events), the results of which are very similar to those of the 13-eruption subset in both PHYDA and CESM. We, nevertheless, control for double events as the most conservative SEA estimate regarding persistence estimates (*SI Appendix*, Fig. S1). For each of the 13 events, the anomalies with respect to a 5-y pre-eruption reference period are calculated for 20 y following the eruption before averaging the response across all 13 events; in the case of double events, the reference period is calculated as the 5 y preceding the first event. Statistical significance of the SEA results is assessed using a Monte Carlo simulation based on the null hypothesis of finding no association between the eruptions and the PDSI anomalies (see *Materials and Methods* for details).

**Fig. 1.**
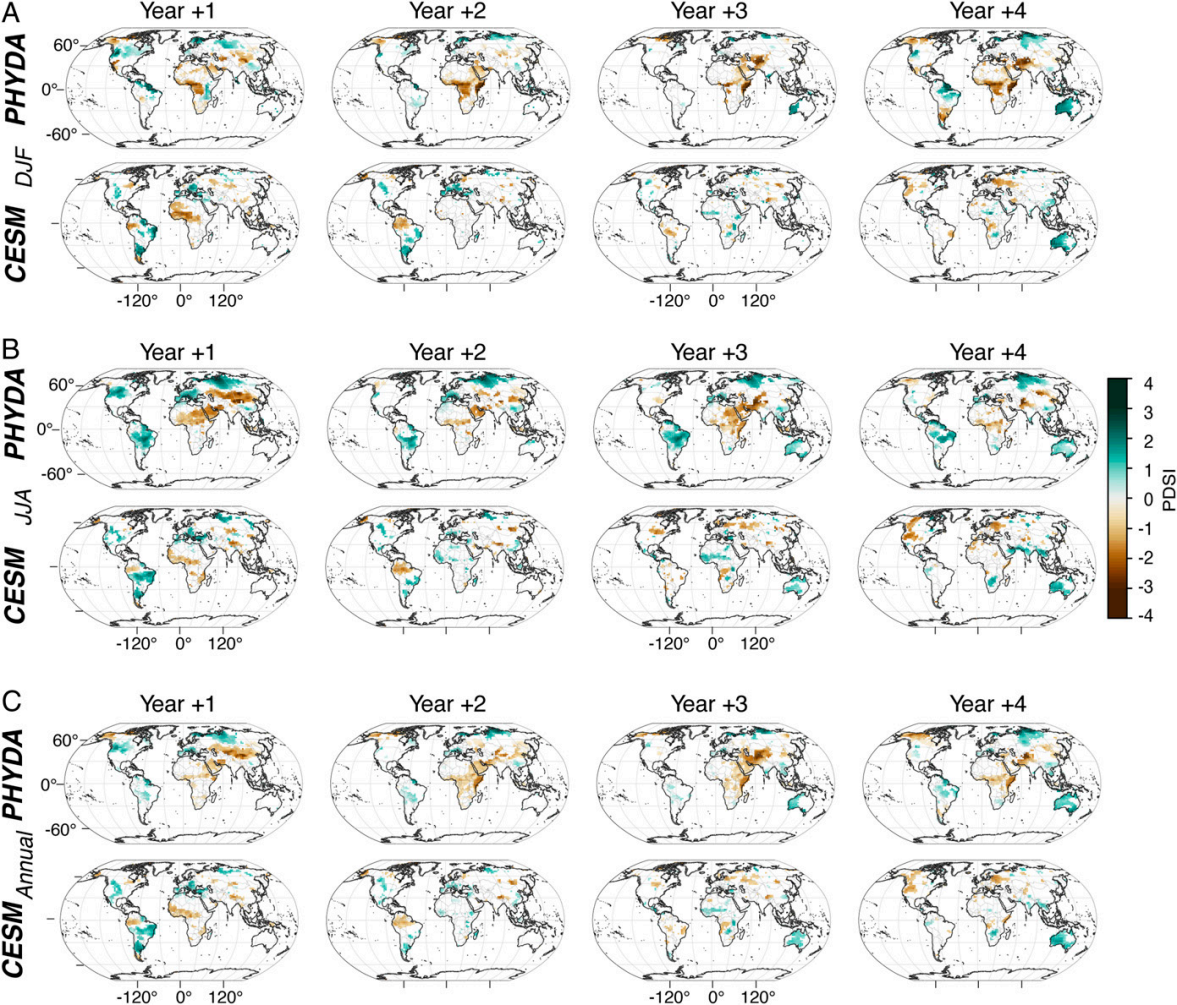
The spatial hydroclimatic response to last millennium large TVEs in PHYDA and CESM. (*A*) Spatial representation of years +1 to +4 of the PDSI SEA analysis for the boreal winter (DJF season) using PHYDA and CESM ensemble member 10. (*B*) As in *A*, except for JJA. (*C*) As in *A*, except for the annual (April through March) season. Only significant values at the 95% confidence level are shown.

**Fig. 2.**
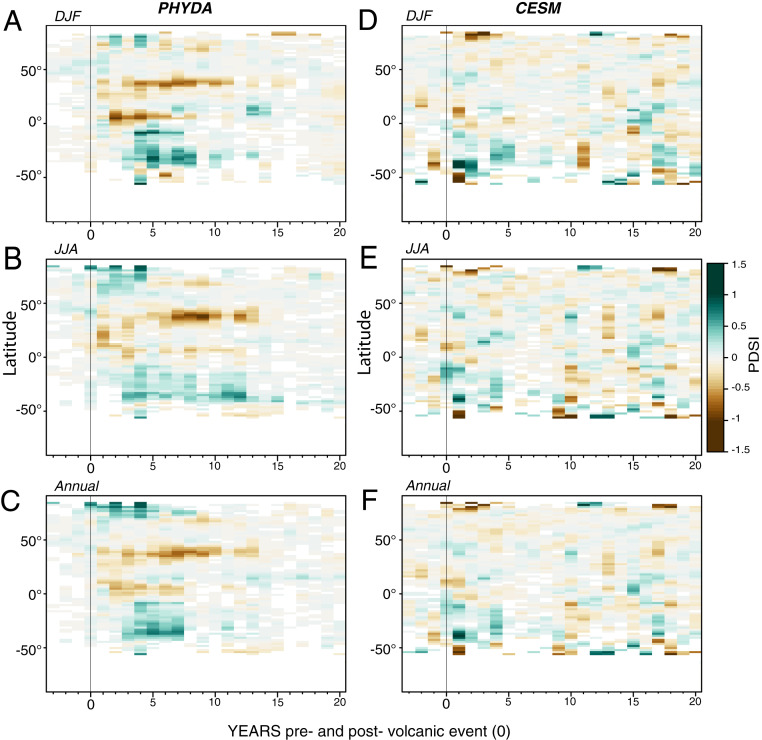
The Hovmöller diagram showing the weighted zonal mean response to TVEP or TVE in different seasons (*A–C*) using PHYDA and (*D–F*) CESM ensemble member 10, respectively. Values that do not satisfy the significance threshold (*P* < 0.05) are excluded. The vertical black line indicates the onset of the volcanic event. Greenland and Antarctica are not included.

We find a significant (*P* < 0.05, *n* = 13) hydroclimatic response in PHYDA following large TVEs (Fig. 1). The hydroclimatic response consists of abrupt regional to continental-scale changes that appear one year following the event. The most notable changes show drying across tropical Africa and into the Middle East and Central Asia, while wetter conditions are evident north of 45°N in the Northern Hemisphere and between ∼5°S and 45°S in the Southern Hemisphere (Fig. 2). The life cycle and radiative forcing after large TVEs (4), which persists for ∼24 months, determines this abrupt climate response, lasting until approximately year +3. However, the responses in multiple regions persist for up to a decade or longer (*SI Appendix*, Figs. S2–S4), suggesting the radiative forcing effects of stratospheric aerosols can influence multiple processes that further modify the atmospheric and oceanic response (5, 33–35). Potential mechanisms for these responses are subsequently reviewed and evaluated against additional evidence from PHYDA-reconstructed variables.

**Fig. 3.**
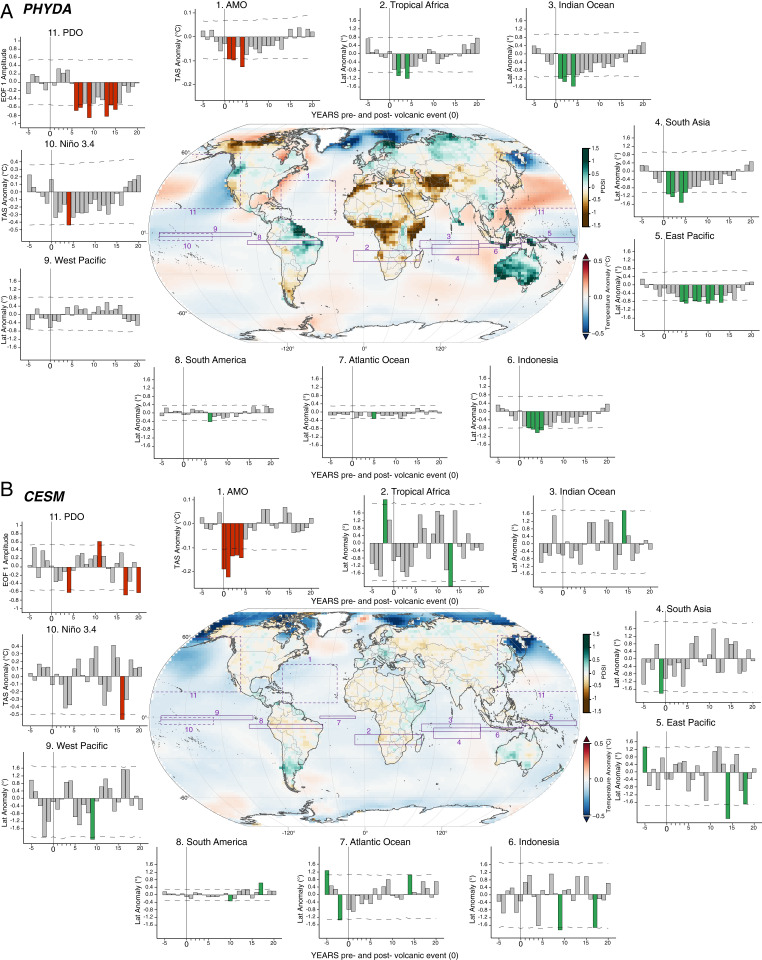
Climate system response in PHYDA and CESM to last millennium large TVEs in the boreal winter. (*A*) Map in center of the panel shows the weighted mean (years +0 to +20) state of the 2-m marine air temperature (ocean) and hydroclimate (land) after TVEP in DJF using PHYDA. Values that do not satisfy the significance threshold (*P* < 0.05) are excluded. The purple boxes in the map indicate the domain of the ITCZ (over ocean)/precipitation (over land), including the extent of the latitudinal shift in DJF. Bar plots (with corresponding numbers) show changes in latitude of ITCZ/precipitation domains from year −5 to +20, with significant changes (at 95% confidence level) colored in green. Dashed purple boxes in the map (numbers 1, 10, and 11) indicate AMO, ENSO, and PDO domains, and the corresponding bar plots show significant changes (at 95% confidence level) in 2-m marine air temperature colored in red. (*B*) As in *A,* except using output from CESM ensemble member 10.

Our initial focus is on ENSO impacts. During the season of peak ENSO influence (December, January, and February [DJF]) (Fig. 3*A*), the persistent cooling in the North Atlantic and tropical Pacific (Atlantic Multidecadal Oscillation [AMO] and Niño 3.4) (Fig. 3*A*) suggests these radiatively induced changes and the subsequent impacts on teleconnection patterns may be a main cause for the extended cooling in PHYDA, which is consistent with previous studies using climate models alone (5, 34). PHYDA estimates that a volcanically induced La Niña–like pattern is most likely to develop in DJF starting in year 1, although it is only significant ∼4 y after the event (Fig. 3*A*), in agreement with previous studies using general circulation models (11, 36) and proxy records (37) but contrary to what some studies using either proxy records alone (38, 39) or models (40, 41) have suggested. The PHYDA-estimated La Niña–like response is nevertheless based on a small number of events, four of which yield an El Niño–like response (1883, 1831, 1815, and 1595) the first year after the eruption. Despite being weak and barely significant, the apparent posteruption cooling in the tropical Pacific does persist for almost a decade in PHYDA and is one potential feedback mechanism for extending radiatively induced cooling by maintaining anomalously cold conditions in the tropical Pacific and the consequent cold extension through the global tropics (42). Additional implications of the La Niña–like state include hydroclimate responses and associated dynamical variable changes, which are largely consistent with a La Niña–like teleconnection pattern (e.g., drying in the American Southwest, southern South America, and tropical Africa and anomalous wet conditions across Oceania and tropical South America) (*SI Appendix*, Figs. S2–S4). Furthermore, the DJF hydroclimatic patterns are consistent with a combination of ENSO and Pacific Decadal Oscillation (PDO) (43, 44) cold phases (Fig. 3*A*), the latter of which is largely reflective of the La Niña patterns already highlighted. A plausible dynamical explanation of the extended global hydroclimatic response is therefore associated with an imposed cold phase in both climatic modes, peaking in year +4 when ∼31% of the global land area is affected by significant dry and wet anomalies, a pattern that is in agreement with ref. 43 over many regions.

The PHYDA estimates of postvolcanic hydroclimatic responses in many cases demonstrate opposite or intensified responses relative to the simulated continental-scale responses in CESM. With the exception of year one, this is true for many of the subsequent years in South America, Africa, and parts of monsoon Asia, while the response over Australia is more consistent between PHYDA and CESM (Fig. 1 *A*–*C* and *SI Appendix*, Figs. S2–S7). While some changes are apparent in JJA (wet anomalies in South America and Europe; drying in Central Africa), the signal in CESM starts to disappear after year four (Fig. 1 and *SI Appendix*, Figs. S5–S7). The magnitude of the wet and dry anomalies in PHYDA is more pronounced spatially and the signal persists longer, in some cases up to 13 y (*SI Appendix*, Figs. S2–S4). An analysis based on time–latitude Hovmöller diagrams highlights this key difference between the two datasets (Fig. 2). The hydroclimate response in PHYDA shows a clear hemispherically asymmetric response with pronounced drying in the northern tropics and wetter conditions south of the equator, persisting for 10 to 13 y in some locations. In contrast, the stand-alone CESM model simulates a much more stochastic response, with little to no persistence of the hydroclimatic signal. Furthermore, the CESM hydroclimate response following the volcanic eruption, when viewed from a zonally averaged perspective, appears to be indistinguishable from the signal prior to the eruption (Fig. 2 *D*–*F*). PHYDA, on the other hand, indicates a clear change in this response in the year following the eruption (Fig. 2 *A*–*C*). This stronger hydroclimate response in PHYDA is consistent with the enhanced cooling of the Northern Hemisphere (*SI Appendix*, Fig. S8), changing the meridional temperature gradient across the two hemispheres and displacing the ITCZ south toward the hemisphere with less cooling (45), where it can reside for several years (Fig. 3*A*). This mechanism is most notable over 1) the Atlantic domain, where the southward ITCZ displacement affects both African and South American monsoons and leads to persistent drying in Central Africa, while the South American monsoon region experiences wetter conditions (Fig. 3*A*); and 2) the Indian Ocean, where the southward ITCZ displacement leads to persistent wet anomalies over Oceania and some areas of Southeast Asia, especially during the DJF season (Fig. 3*A*). On the other hand, the much weaker and less persistent hydroclimate response to volcanic forcing in CESM (Figs. 2 and 3*B*) is not entirely unexpected, at least in the tropics, given the more uniform cooling across hemispheres relative to PHYDA (*SI Appendix*, Fig. S8), which causes only short-lived or nonexistent displacements of the ITCZ (Fig. 3*B*). Finally, we note that some of the changes evident in Fig. 3 may not require dynamic feedbacks per se but may simply generate extended persistence because of the large heat capacity of water and thus ocean temperature changes that persist longer than land temperatures. The response of the AMO, for instance, may simply reflect this heat capacity difference, which may, in turn, contribute to some of the hydroclimatic anomalies over land adjacent to the Atlantic.

## Discussion

**Fig. 4.**
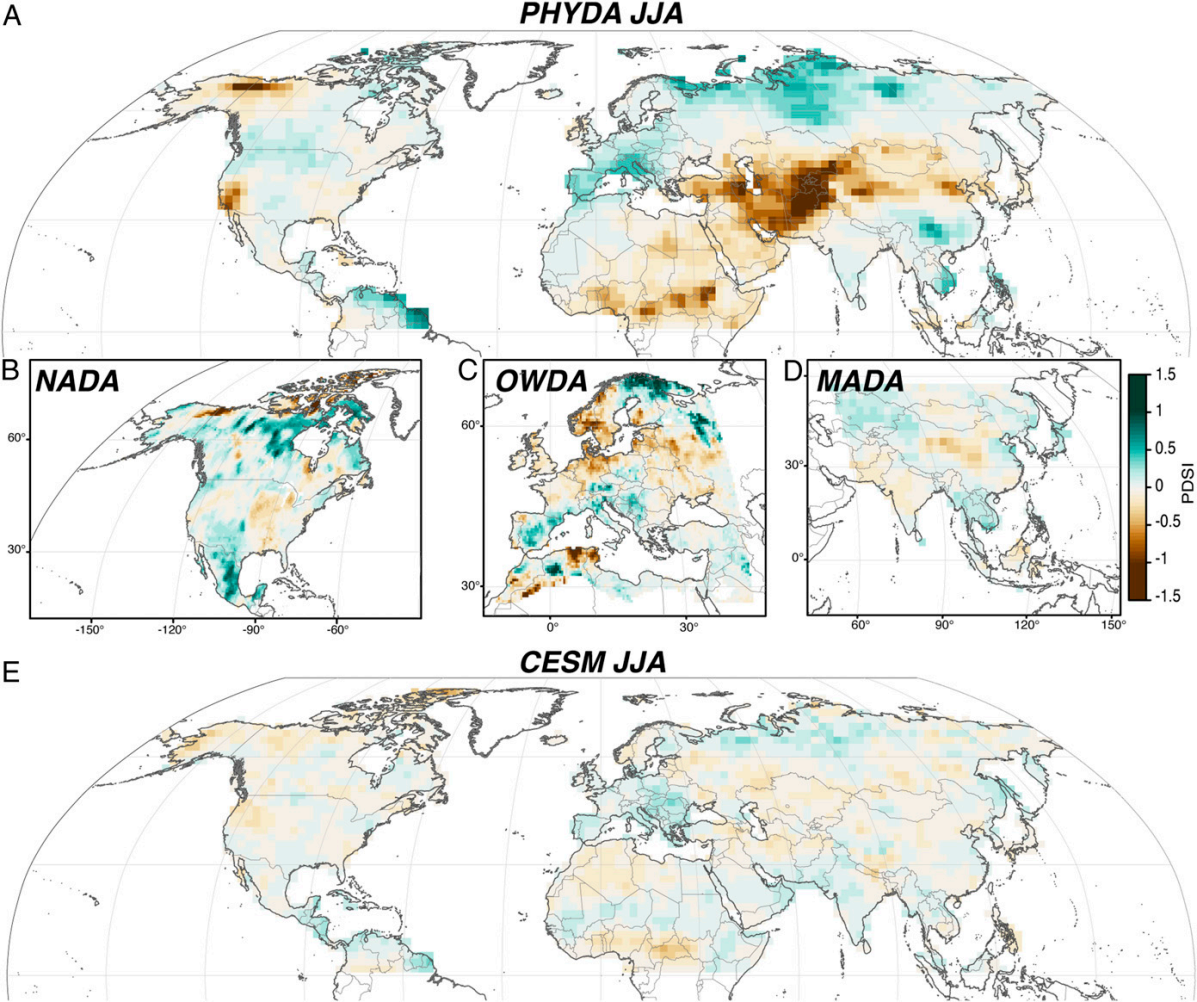
A comparison of JJA hydroclimatic responses estimated from PHYDA and drought atlases in the Northern Hemisphere. (*A*) The weighted mean (years +0 to +20) state of Northern Hemisphere hydroclimate after TVEP in JJA using PHYDA. (*B*) As in *A*, but using the NADA (49) and domain instead. (*C*) As in *A*, but using the OWDA (48) and domain instead. (*D*) As in *A*, but using the MADA (50) and domain instead. (*E*) As in *A*, except for CESM ensemble member 10. Note that MADA starts in 1300 CE, and thus only seven events were included in the SEA in *D*. Values that do not satisfy the significance threshold (*P* < 0.05) are excluded.

PHYDA has been extensively validated against observational data, paleoclimate reconstructions (29), and in comparisons to model experiments (46, 47). Fig. 4 provides comparisons between the volcanic responses in PHYDA, the Old-World Drought Atlas (OWDA) (48) over Europe (22, 23), and the North American Drought Atlas (NADA) (49). These latter reconstructions rely on higher-resolution datasets for the JJA season and do not employ climate model information; comparisons between the drought atlases and PHYDA indicate that there are unresolved uncertainties in the proxy estimates. Both PHYDA and OWDA responses include decadal-scale wet anomalies in southern and northeastern Europe, although there is no significant drying over northwestern Europe in PHYDA. These differences are likely the product of 1) the lower spatial resolution of PHYDA; 2) OWDA including a different proxy network, especially over central Europe; and 3) the different spatial covariance assumptions in the two reconstruction methods (i.e., the localized spatial covariance approach applied in the point-by-point regression method used to produce the drought atlases and the model-informed spatial covariance inherent to PHYDA). Over North America, both PHYDA and NADA show drying in the southeastern United States, wetter conditions in the central United States and Canada, and drying over northern Alaska, although a much stronger drying signal emerges over the southwestern United States in PHYDA. While the patterns across these three paleoclimatic datasets do indicate existing uncertainties, they are nevertheless consistent in regard to the persistence and magnitude of the hydroclimatic anomalies that they represent. In other words, the hydroclimatic responses in OWDA, NADA, and PHYDA are of similar magnitude when averaged over the 20 y following an eruption, while the CESM response—in contrast to the proxy-based datasets—is characterized by much more muted multidecadal responses.

Over Asia, the JJA hydroclimatic response to volcanic eruptions has been analyzed previously, based on both tree-ring data (24, 25) and the CESM-LME (9, 13), with diverging results. Our results indicate wetter conditions following eruptions over southern continental Asia alongside a drier climate over Indonesia. While PHYDA is more consistent with estimates from the tree-ring based Monsoon Asian Drought Atlas (MADA) (50) than the CESM volcanic estimate, PHYDA and MADA are not well correlated over much of their overlapping domains, and the magnitude of the MADA response is more muted. These differences are likely due to the small number of proxy records over monsoon Asia relative to the proxy densities in the OWDA and NADA regions and the comparatively poor instrumental data quality over the MADA domain that impacts the robustness of proxy calibrations (29, 50), both of which increase reconstruction uncertainties in the MADA and PHYDA. Furthermore, the comparison is complicated by the fact that MADA only reaches back to 1300 CE, and hence, only seven volcanic events are included in the MADA SEA, as compared to 13 in the PHYDA analysis. An analysis (*SI Appendix*, Fig. S9) using only the subset of seven MADA events indicates that the inconsistencies between PHYDA, MADA, and CESM are not resolved by correcting for sampling alone, suggesting that significant uncertainties in the proxy-estimated hydroclimatic response are still unresolved.

Even though we compare PHYDA to the same ensemble member used as the data assimilation prior (see *Materials and Methods*), the general results of our comparisons are not dependent on the employed CESM-LME member. The hydroclimatic response to volcanic eruptions is equally muted, relative to PHYDA, in the other available ensemble members of the CESM-LME (*SI Appendix*, Figs. S10 and S11); this result is likely because we focus on very large radiatively forced responses and composite across 13 events in each ensemble member. Our general conclusions, therefore, are not dependent on a single ensemble member of CESM, although some specifics of our findings like the ENSO responses sometimes varies across ensemble members because of how the tropical Pacific is randomly preconditioned. The main differences between PHYDA and CESM may, therefore, arise from structural components of the CESM model (such as how radiative perturbations are implemented in the simulations (e.g., refs. 4 and 13), inaccurate estimates of volcanic forcing (see *Materials and Methods*), or uncertainties in the climatic response of proxies or their interpretation in PHYDA. If the origins of these differences are associated with modeling choices, however, our findings suggest that risk assessments of volcanic eruptions that are based solely on ESMs may be subject to significant biases, although a more comprehensive analysis over an ensemble of ESMs is important before larger conclusions can be drawn (16).

Uncertainties in PHYDA are typical of most paleoclimatic studies. Proxy sampling inhomogeneities in both time and space may affect the nature of the PHYDA reconstruction. For example, in the Southern Hemisphere (SH), where fewer proxies are available, the model prior may contribute more to the character of the estimated field. A more muted SH response therefore may not only result from physical processes but, to some extent, also reflect proxy availability. We note, however, that the signal in the SH is different in PHYDA and the CESM-LME, arguing in favor of added information from the assimilated proxy data. There are also uncertainties in the timing and magnitude of the volcanic events as estimated from ice cores. The volcanic dataset used herein is considered to have the most precise available reconstruction but still has an average precision of ±2 y during the past 1,500 y (1). These uncertainties could, therefore, impact the PHYDA interpretation by causing the estimate of year zero in the SEA composite to be incorrectly selected; this dating uncertainty is irrelevant for the model simulations in which the imposed year of the eruption is known by construction. The impacts of these uncertainties on the PHYDA–CESM-LME comparison have several implications that may increase or decrease their described differences. Regarding the magnitude of the volcanic responses, dating errors would uniformly reduce the PHYDA-estimated magnitudes because the composite will include some years that are either before or after the true eruption year and thus smear out the largest response across the SEA. In regard to persistence, however, dating errors that select years before the actual eruption will increase the persistence estimates, while dating errors that select years after the actual eruption will reduce the persistence estimate; thus, the net impact will ultimately depend on the collective number of dating errors in either direction.

Recent investigations have also noted the impact of biological persistence on temperature reconstructions derived from tree-ring width (TRW) (e.g., refs. 17 and 18), which can artificially enhance the persistence of cooling estimates tied to volcanic eruptions. Although recent studies have focused on temperature, biological persistence can also impact hydroclimatic reconstructions (e.g., refs. 51 and 52). Maximum latewood density (MXD) has been proposed as a proxy that avoids the impacts of biological persistence and therefore constitutes a better tree-ring proxy for estimating temperature responses to volcanism. An MXD-only and a mixed MXD–TRW reconstruction of Northern Hemisphere temperatures have shown that the persistence of volcanic cooling is reduced relative to reconstructions that rely exclusively on TRW (18, 21). Northern Hemisphere SEA estimates from these two reconstructions are compared to the PHYDA estimate in *SI Appendix*, Fig. S12, clearly showing similar responses to volcanic cooling in the PHYDA and MXD-only reconstructions (both of which compare well to the CESM ensemble), while the mixed MXD–TRW reconstruction is the most persistent proxy estimate. These comparisons provide strong evidence that the PHYDA agrees well with state-of-the-art temperature reconstructions that have sought to remove the effects of biological persistence. Despite relying heavily on TRW chronologies, these favorable findings are likely in part due to the multiproxy nature of the PHYDA methodology. It is also worth noting that while biological persistence in TRW has recently been a topic of renewed interest in the volcanic literature, much of the original work on the issue focused on hydrological reconstructions (e.g., ref. 52). This early work has led to applications of signal processing techniques that have been developed to correct for nonclimatic persistence in dendroclimatic chronologies (e.g., ref. 51). These techniques are now widely applied and were used to construct many of the chronologies that comprise the PHYDA proxy network. Such considerations, along with the results we present in *SI Appendix*, Fig. S12, thus provide strong evidence for the robustness of the PHYDA persistence estimates, although persistence biases in the PHYDA estimate cannot be completely ruled out. Finally, we note that PHYDA also estimates much larger hydroclimatic responses than CESM (in addition to different spatial patterns). We are not aware of any obvious proxy process that would artificially enhance the magnitude of the estimated hydroclimatic response, leaving the differences between the severity and spatial patterns in PHYDA and CESM hard to explain based on proxy uncertainties alone.

Our analyses suggest that TVEPs (>∼8 Tg S) cause significant hydroclimatic impacts over large continental regions. The number of available events is too small to robustly associate the magnitude of the eruption with the size and persistence of the impacts, which are ultimately determined by event-specific factors, such as the composition of the ejecta and the volume of aerosols that is ultimately emplaced in the stratosphere. The described impacts nevertheless represent risks associated with volcanic events that, based on the volcanic record of the last millennium, are likely to happen in any given century, including the present one. Notable impacts include significant drying over tropical Africa and the Middle East and Central Asia and wet anomalies over much of Oceania and the South American monsoon region. These changes represent statistically significant anomalies that persist for more than a decade in some regions. The persistence of the anomalies is associated with southward shifts in the ITCZ and SST changes in the Pacific and Atlantic oceans. Even in the absence of global warming, a decade of dry or wet anomalies after a large TVE could have huge socioeconomic implications for many locations around the world. For instance, the greater Middle East, which already is experiencing a general drying trend and includes 29 countries and 550 million people (53), is estimated by PHYDA to be subject to significant decadal drying, with associated regional impacts and risks (e.g., in the availability of fresh water, agricultural losses, and energy systems). In Sub-Saharan Africa, with a population of over a billion people that includes some of the communities most vulnerable to climate change (54), direct impacts of persistent drying, such as those estimated by PHYDA after large TVEs, would have large impacts on food and water security (55). Finally, western North America has experienced a drying trend since the late 1990s, the dramatic socioeconomic impacts of which have served as a catalyst for the creation of management institutions and the implementation of sophisticated long-term measures to mitigate the impact of future droughts (56–58). This region is similarly at risk for decadal-scale drying after large volcanic eruptions, again illustrating the compounding risks of climate change and volcanism. Our proxy-based analysis and the associated comparisons with the CESM-LME therefore highlight the critical importance of further characterizing the hydroclimatic responses to volcanism and reconciling their representation in both large-scale proxy reconstructions and model simulations.

## Materials and Methods

### Climate Data.

The PHYDA is a publicly available global reconstruction of hydroclimate, surface temperature, and associated dynamic climate variables over the past 2,000 years (29). PHYDA combines 2,978 seasonally or annually resolved proxy time series (2,591 tree-ring records, 197 coral and sclerosponge records, 153 ice core isotope records, 26 speleothem isotope records, 10 lake sediment records, and one marine sediment record) with the physical constraints of an atmosphere-ocean climate model [the CESM-LME simulation number 10 (59)]. Our principal analysis is a comparison between the PHYDA and the same ensemble member used as the prior, ensuring that the differences in the estimated responses are a result of the proxy information that is assimilated in PHYDA. A comparison between PHYDA and all members of the CESM-LME is nevertheless also explored by additionally analyzing ensemble members two through nine (*SI Appendix*, Figs. S10 and S11).

We use a 100-member PHYDA ensemble, randomly selected from the full 1,000-member ensemble, to provide robust uncertainty estimates for the derived reconstruction. We limit our analysis to the 1000 to 1850 CE interval, as the uncertainty of the reconstruction significantly increases prior to 1000 CE in many regions. We analyze annual (defined as April to March of the next calendar year), boreal summer (JJA), and austral summer (DJF) means. We employ the PHYDA-reconstructed 2-m surface temperature (only over the ocean) and the PDSI (only over land), both of which are reconstructed on approximately a 2° latitude-longitude grid. We additionally use several PHYDA-reconstructed dynamical variables in each season: the North Atlantic SST index, which is the nondetrended and nonsmoothed version of the AMO; the monthly Niño 3.4 index; and the latitude of the ITCZ in eight longitudinal zones (ITCZ positioning in PHYDA was reconstructed from indices calculated for the CESM model field based on a precipitation center of mass formulation as described in refs. 29 and 60). In total, 13 output variables from PHYDA were analyzed (*SI Appendix*, Table S1). Anomalies of temperature (in °C) and ITCZ location (in degree of latitude) were calculated with respect to 1000 to 1850 CE. Normalized values of PDSI were used as reconstructed in PHYDA. Finally, we calculated the PDO for DJF as the first empirical orthogonal function of the PHYDA-reconstructed surface air temperature over the Pacific Ocean north of 20°N (61).

We use the same PHYDA-selected variables from the CESM-LME and perform the equivalent analyses with the model data. Model PDSI was computed using the Penman-Monteith equation for potential evapotranspiration and monthly climate model output of precipitation, 2-m air temperature, vapor pressure, net surface radiation, surface pressure, and surface wind (estimated from 10 m down to 2 m using the wind profile power law); the climatologically bias-corrected temperature and precipitation fields were used in the calculations. PDSI was computed using the MATLAB code from ref. (62), which produced the standard formulation of PDSI as opposed to self-calibrating versions (e.g., ref. 63).

PHYDA was constructed as an offline data assimilation product in which the model prior is the same for each year and is drawn from all years in an existing climate model simulation (i.e., the ensemble members in the prior are seasonally or annually averaged climate states from one simulation instead of an ensemble of independently running model simulations), as in traditional online data assimilation. One implication of this approach is that while the model simulation that comprises the prior has an explicit temporal history tied to its forcing data, the timing of climate events such as volcanic eruptions or trends over specific periods (e.g., twentieth century warming) are not dictated by the prior. Consequently, all temporal structure in the PHYDA product is tied specifically to information contained in the assimilated proxy network (29).

The largest TVEs of the twentieth century (Agung 1963, El Chichón 1982, and Mount Pinatubo 1991) coincided with prevailing El Niño conditions (64). Some studies analyzing the impacts of volcanic events on climate have thus suggested the need to remove the ENSO signal prior to the analysis by either detrending the data (19) or considering the residuals after regressing the data against ENSO (25). Here, we did not remove the ENSO signal from the variables studied, as the median ensemble value of the PHYDA Niño 3.4 index in the year preceding each selected eruption was close to zero and thus indicates that neutral conditions were the dominant ENSO state prior to the analyzed eruptions (*SI Appendix*, Fig. S13). Potential effects of longer-term changes in the mean state were removed by constraining the reference period of the SEA to the last 5 y before an event.

### Volcanic Forcing Index.

We use the most recent global volcanic forcing reconstruction based on a suite of ice core records from Greenland and Antarctica, named “eVolv2k_v2” (1). This database contains estimates of the magnitudes and approximate source latitudes of major VSSI events from 500 BCE to 1900 CE. We employ this data product instead of the earlier reconstructions (31, 32, 65) because of significant improvements in the ice record (regarding dating and synchronization) and refinements to the methods used to derive VSSI estimates. Accurately selected eruption dates are crucial for performing the SEA on the proxy data and thus obtaining a precise picture of the global climate response to large volcanic events.

We select those volcanic events located within the tropics (+25° N to −25° S) with magnitudes greater than Mount Pinatubo [VSSI > 8.78 Tg S (1)] from 1000 CE to 1850 CE, herein referred to as TVEP. The focus on tropical eruptions is motivated by the known climatic signal of large TVEs, related to seasonal variations in the ITCZ, which facilitates the transfer of aerosols between hemispheres (66, 67). We note, however, that while our selection process is consistent with the state-of-the-art selection procedures in the literature, all eruptions are not created equal and the eruption magnitude estimates do not imply proportional deposition of aerosols in the stratosphere, nor is the composition of the deposits the same for every eruption. Climatic responses to the eruptions therefore cannot be assumed to scale proportionally with the estimated magnitude of the eruptions. To avoid potential biases induced by “double event” occurrences, we selected only the second event when two events occurred within 10 y (e.g., we included the event in 1458 but not the one in 1453; see *SI Appendix*, Fig. S14). To perform the same analysis using CESM, we select the 13 equivalent volcanic events as in ref. (1) because those dates and estimates (31) were used to force the CESM runs. For those cases in which the dates do not align between the two volcanic forcing reconstructions, we select the closest date to the ref. (1) estimated event. These 13 events simulated by CESM are referred to as large TVEs. The 13 selected TVEP and TVE and their characteristics are listed in *SI Appendix*, Table S2. We have also performed the same analysis in CESM using a selection of the volcanic events larger than the 1991 Pinatubo eruption exclusively using the ref. (31) volcanic reconstruction (in contrast to selecting events in this reconstruction that are adjacent to the selected eVolv2k_v2 events). The analysis yields 12 events, 8 of which were included in the 13-event eVolv2k_v2 selection, and 4 of which are excluded due to our double event restriction. An analysis of these events and comparison to the results that we have presented based on the eVolv2k_v2 selection demonstrates a strong consistency between the two selection approaches, with only a small intensification in the CESM results if the selection is exclusive to the ref. (31) reconstruction (*SI Appendix*, Figs. S15 and S16).

### Analysis Methods.

The impacts of volcanic events on global climate were isolated using SEA (30). SEA has been widely used to detect and quantify the climate response to particular volcanic events (e.g., refs. 17 and 20). The method involves sorting data into categories dependent on a key date (volcanic events; *SI Appendix*, Table S2). For each category, the year of the eruption is assigned as year 0, and the values of each analyzed variable (temperature, PDSI, and dynamical variables) are extracted for the 5 y prior to the eruption and for 20 y posteruption to obtain a SEA matrix (number of volcanic events × 26). For each event, the anomalies with respect to the pre-eruption reference period (averaged over years −5 to −1) are calculated, before averaging the response across all 13 events, thereby obtaining a composite of all the events for the 26 y. The short reference period of only 5 y was chosen to avoid combining events into a composite that is affected by changes in the mean background state as a result of low-frequency changes during the last millennium; the SEA reference period in double event cases is taken as the 5 y preceding the first event.

To test for statistical significance of the SEA results, we applied a Monte Carlo simulation based on the null hypothesis of finding no association between the eruptions and the climatic variables studied (i.e., no significant difference in the variables during the years following eruptions, when compared to noneruption years). Random years are chosen as pseudo-event years for each approach (TVEP and TVE) (*SI Appendix*, Table S2), after which the average values for years −5 to +20 are calculated in the same way that was done for real eruptions. This process is repeated 10,000 times to create 10,000 randomly generated composite matrices. Finally, a random composite distribution is created for each column in the matrix (i.e., for each year from year −5 to year +20, relative to the eruption year 0). The distributions are then used to statistically compare the existing composites. We used these distributions to test the significance of the actual composites at the 95% confidence level. To assess the spatial global distribution of the climatic effects, this method is applied to all grid cells included in the 100-member PHYDA ensemble (i.e., each ∼2 degree grid cell is treated as an independent reconstruction, yielding a total of 13,824 grid cells). The same process is applied to each ensemble member of the CESM-LME, including the same number of grid cells. We remove Greenland and Antarctica from the SEA analysis of PDSI because the PDSI is meaningless over a land surface covered by ice. To highlight the persistence of the significant hydroclimatic changes over land, we computed the mean for each cell from years 0 to +20 after the volcanic eruption; values that do not satisfy the significance threshold (*P* < 0.05) were excluded.

## Supplementary Material

Supplementary File

## Data Availability

The PHYDA dataset is available via Zenodo web repository (https://zenodo.org/record/1198817). The “reconstructed volcanic stratospheric sulfur injections and aerosol optical depth, 500 BCE to 1900 CE, eVolv2k_v2” is available via Deutsche Klimarechenzentrum web repository (https://cera-www.dkrz.de/WDCC/ui/cerasearch/entry?acronym=eVolv2k_v2). The processed data fields that are the product of our analysis are available via Zenodo web repository (https://doi.org/10.5281/zenodo.4543919).
