# Supplementary material for: Global hydroclimatic response to tropical volcanic eruptions over the last millennium
Source: Proc Natl Acad Sci U S A. 2021 Mar 8;118(12):e2019145118. doi: 10.1073/pnas.2019145118 (PMC8000584; doi:10.1073/pnas.2019145118)
Supplement: Supplementary File [file pnas.2019145118.sapp.pdf]

PNAS
www.pnas.org

Supplementary Information for

Global hydroclimatic response to tropical volcanic eruptions over the Last Millennium

Ernesto Tejedor; Nathan J. Steiger; Jason E. Smerdon; Roberto Serrano-Notivoli; Mathias Vuille

Ernesto Tejedor
Email: etejedor@albany.edu

This PDF file includes:

Figures S1 to S16
Tables S1 to S2
References

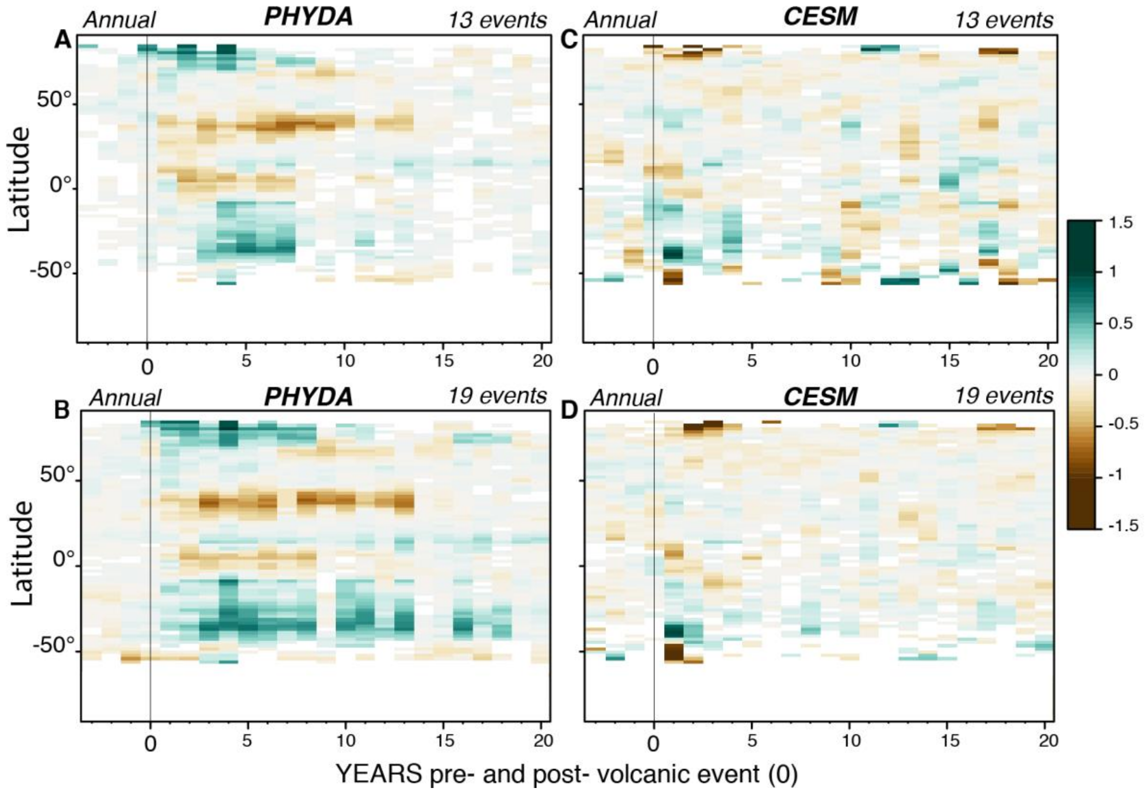

Fig. S1. Hovmöller diagram of the hydroclimatic response for the annual season. Hovmöller diagram showing the weighted zonal-mean hydroclimatic (PDSI) response to volcanic eruptions for the annual season using (**A**) PHYDA and TVEP, excluding double-events, (**B**) PHYDA and TVEP, including all events, (**C**) CESM ensemble member 10 and TVP excluding double-events, (**D**) CESM ensemble member 10 and TVP including all events. Values that do not satisfy the significance threshold ($p<0.05$) are excluded. Vertical black line indicates the onset of the volcanic event. Greenland and Antarctica are not included.

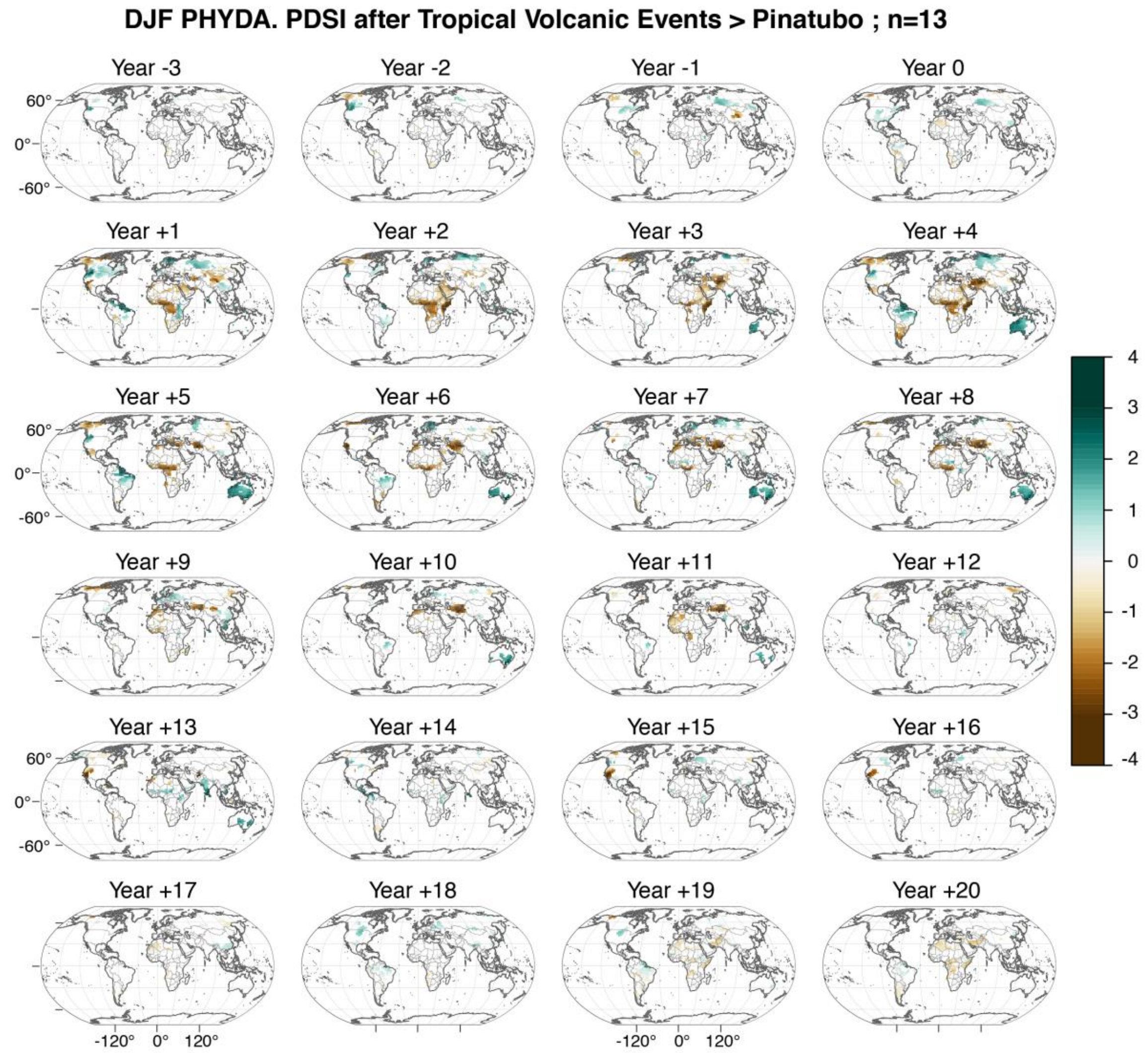

Fig. S2. Global DJF hydroclimatic response to TVEP in PHYDA. Global DJF hydroclimatic response to Last Millennium large tropical volcanic events using PDSI estimated from PHYDA (n=13, see Methods). SEA performed for 20 years with respect to the 5-year mean before the event (year 0). Only significant values at the 95% confidence level are shown.

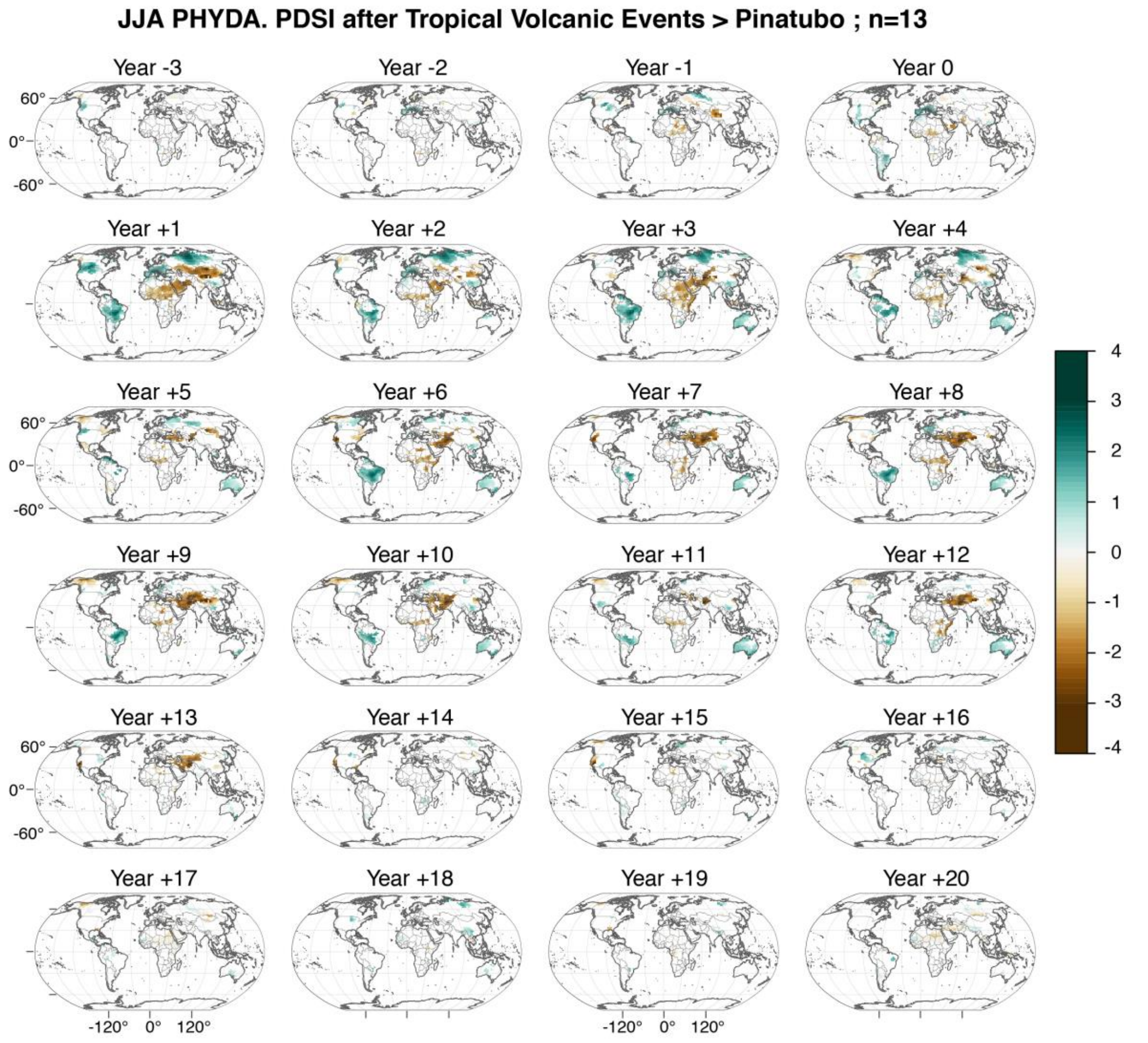

Fig. S3. Global JJA hydroclimatic response to TVEP in PHYDA. Same as in fig. S2, but for JJA.

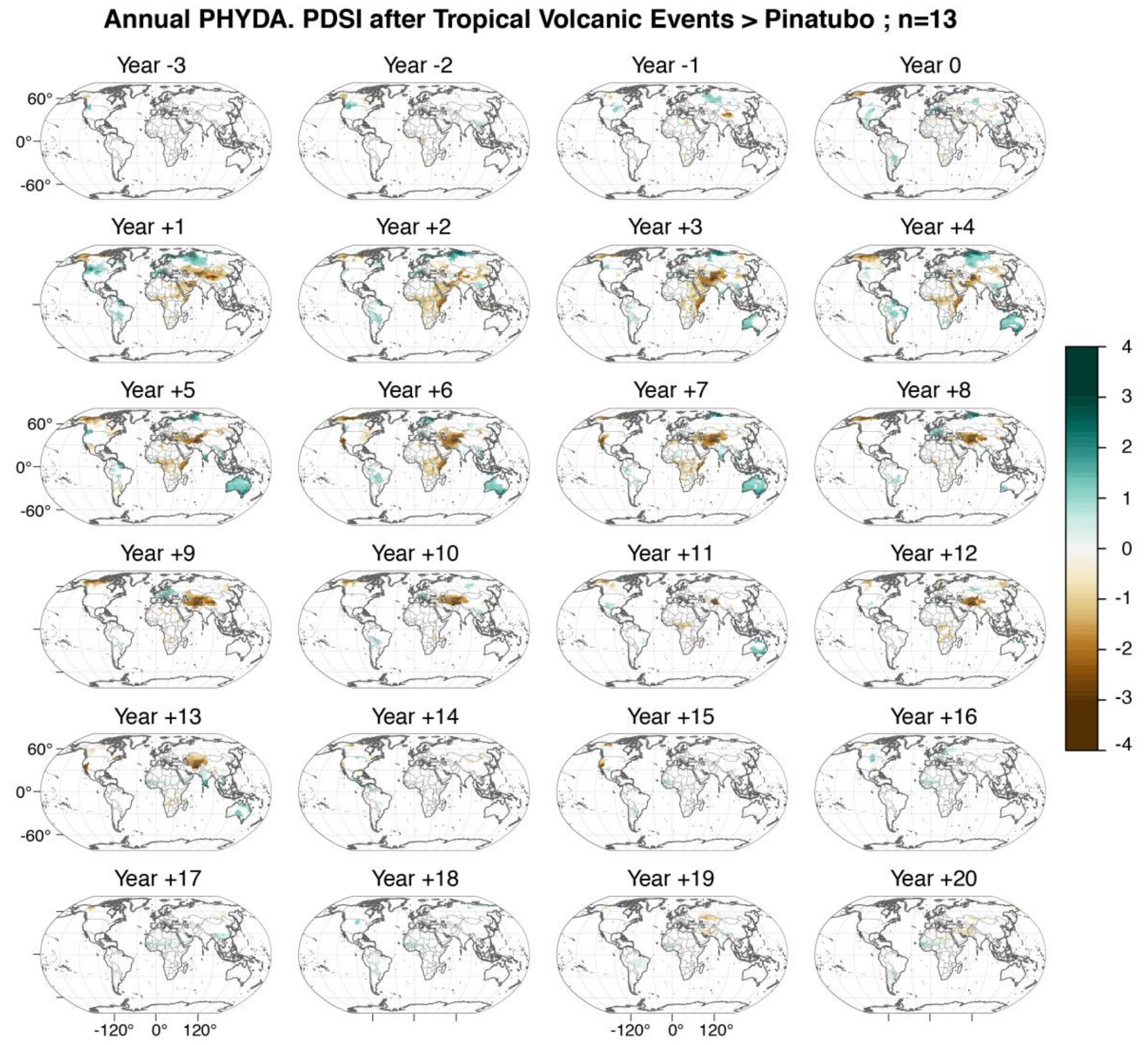

Fig. S4. Annual hydroclimatic response to TVEP in PHYDA. Same as in fig. S2, but for the Annual (Apr.-Mar.) field.

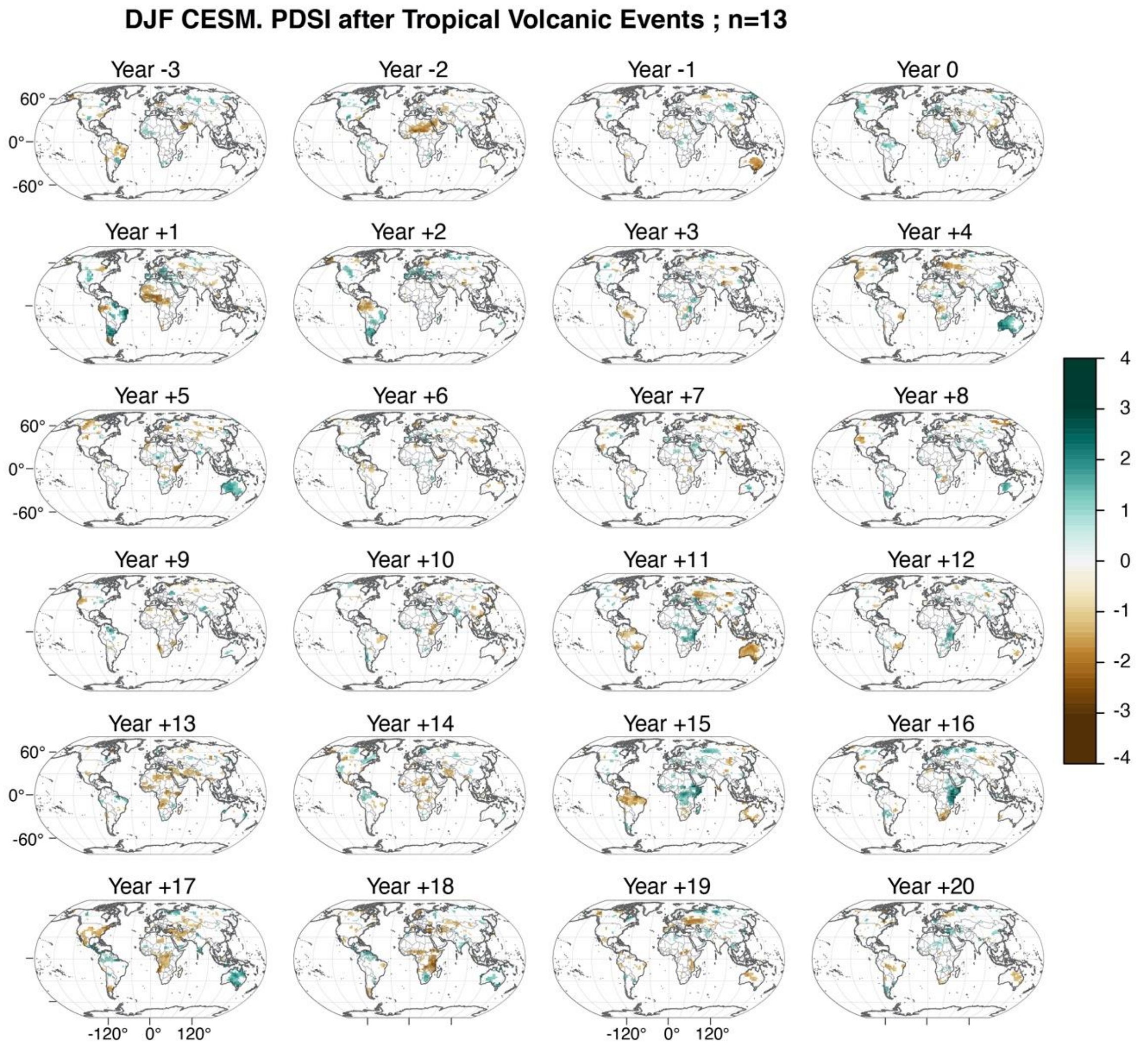

Fig. S5. Global DJF hydroclimatic response to TVE in CESM. Global DJF hydroclimatic response to Last Millennium large tropical volcanic eruptions (equivalent to Toohey et al., 2017, but based on Gao et al., 2008) using PDSI calculated from CESM ensemble member 10. SEA performed for 20 years with respect to the 5-year mean before the event (year 0). Only significant values at the 95% confidence level are shown.

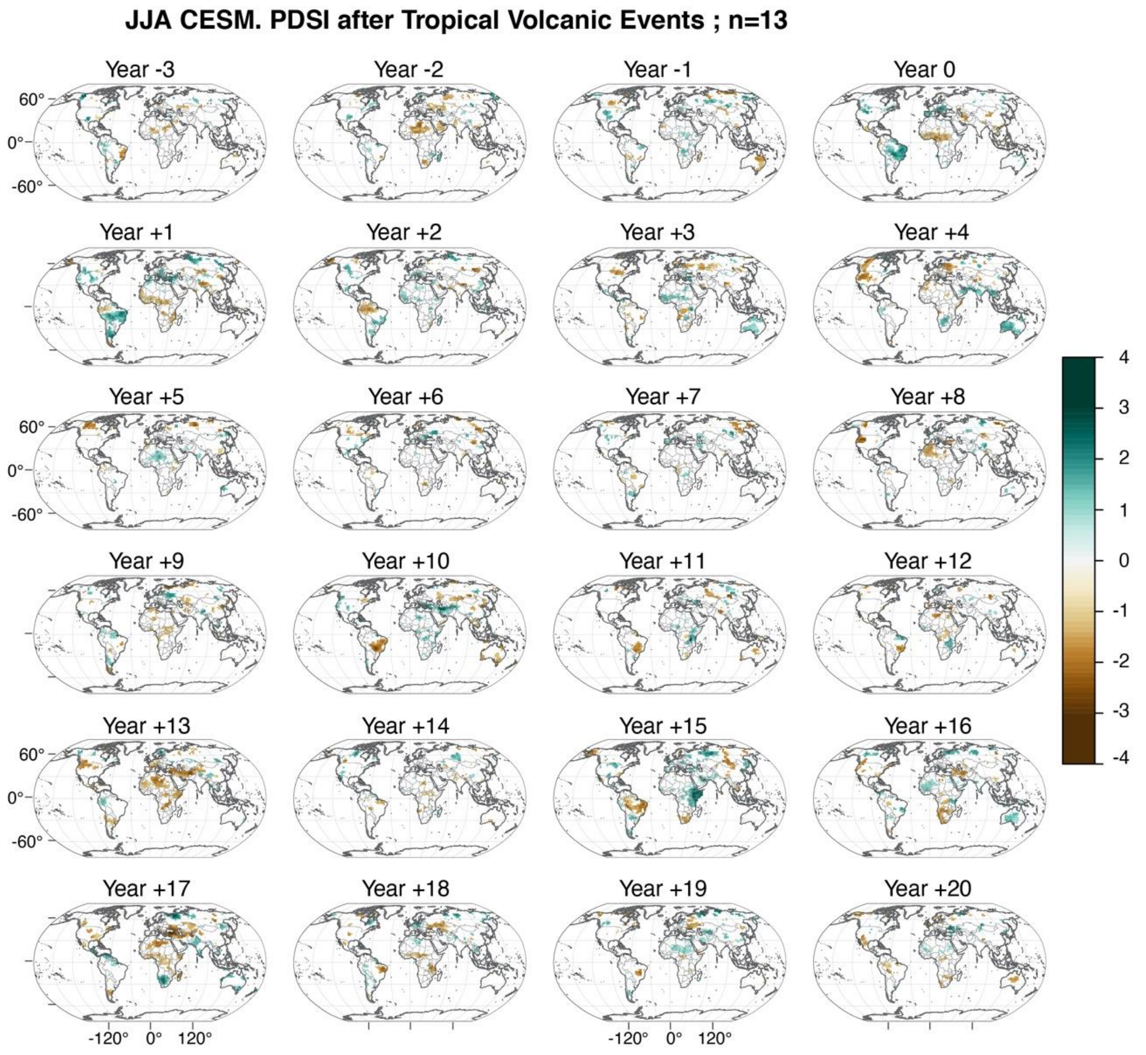

Fig. S6. Global JJA hydroclimatic response to TVE in CESM. Same as in fig. S5, but for JJA.

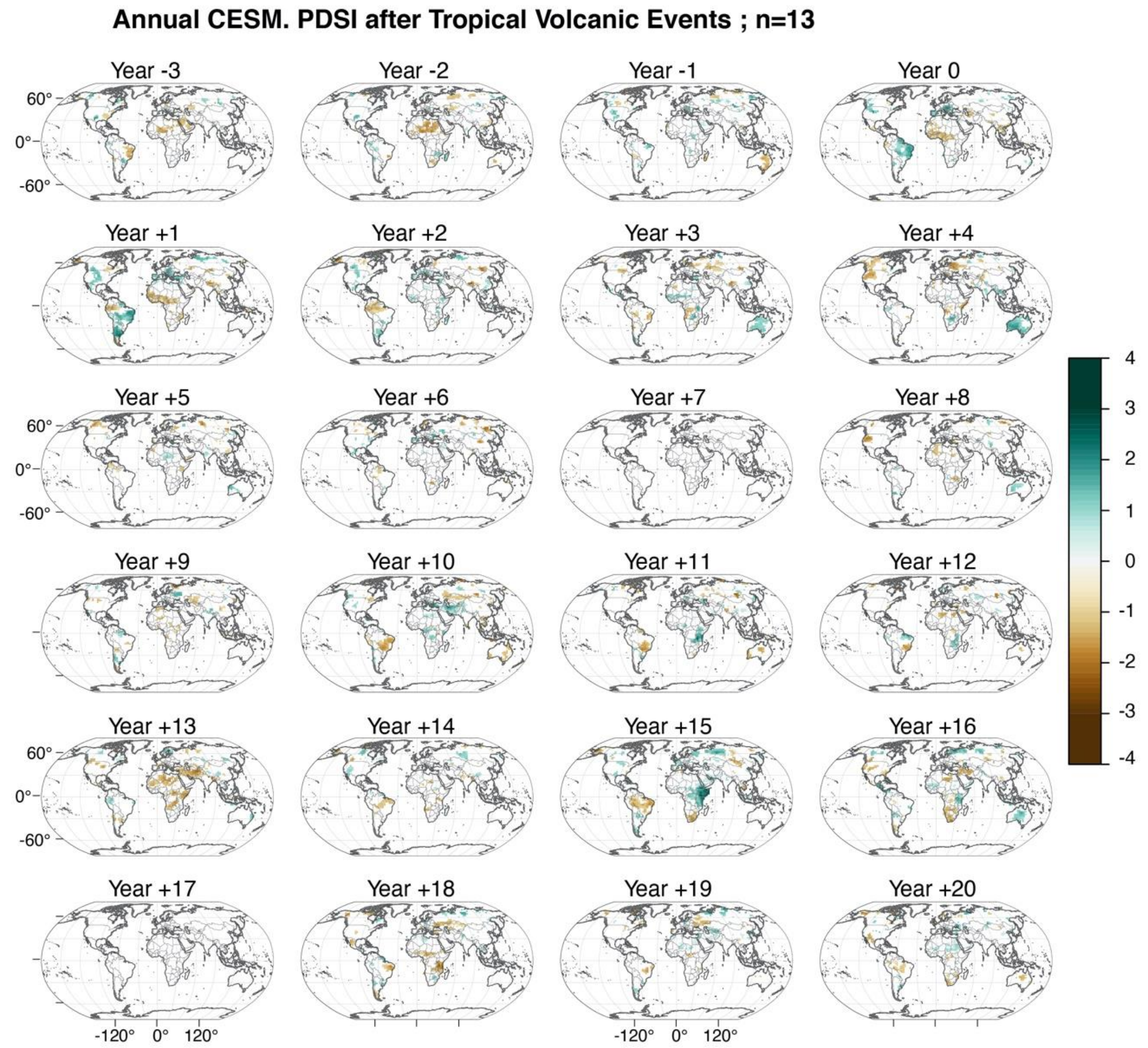

Fig. S7. Global Annual hydroclimatic response to TVE in CESM. Same as in fig. S5, but for the Annual (Apr.-Mar.) field.

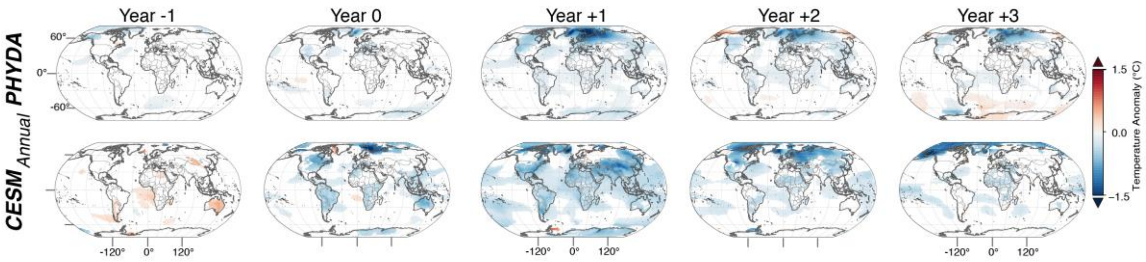

Fig. S8. Temperature response to TVEP and TVE in PHYDA and CESM. Spatial temperature response to Last Millennium large tropical volcanic events in PHYDA and CESM ensemble member 10. Spatial representation of the years -1 to +3 of the temperature (in ºC) SEA analysis for annual resolution using PHYDA and CESM. Only significant values at the 95% confidence level are shown. The maps show the enhanced cooling of the Northern Hemisphere in PHYDA changing the meridional temperature gradient across the two hemispheres and displacing the ITCZ south toward the hemisphere with less cooling, as opposed to the case of CESM, where a more uniform cooling across hemispheres does not lead to coherent ITCZ displacements.

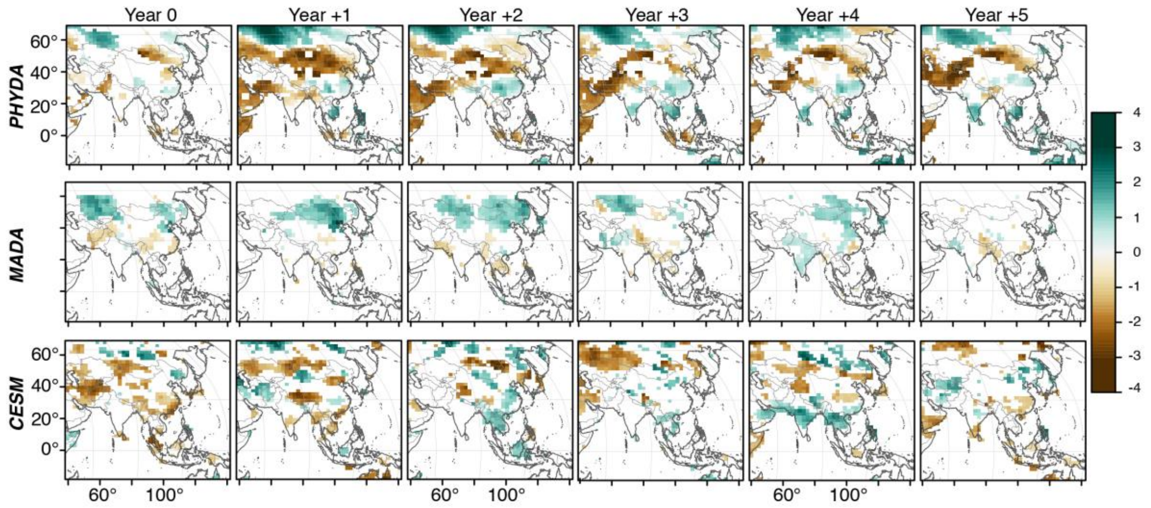

Fig. S9. Hydroclimatic response to TVEP in PHYDA, MADA and CESM. Hydroclimatic response (PDSI) in PHYDA, MADA and CESM ensemble member 10 to Last Millennium large tropical volcanic events (7 events larger than Mt Pinatubo 1991) in JJA since 1300 CE (spanning the length of MADA's reconstruction). SEA performed for 20 years (only the first +5 years are shown) with respect to the 5-year mean before the event (year 0). Only significant values at the 95% confidence level are shown.

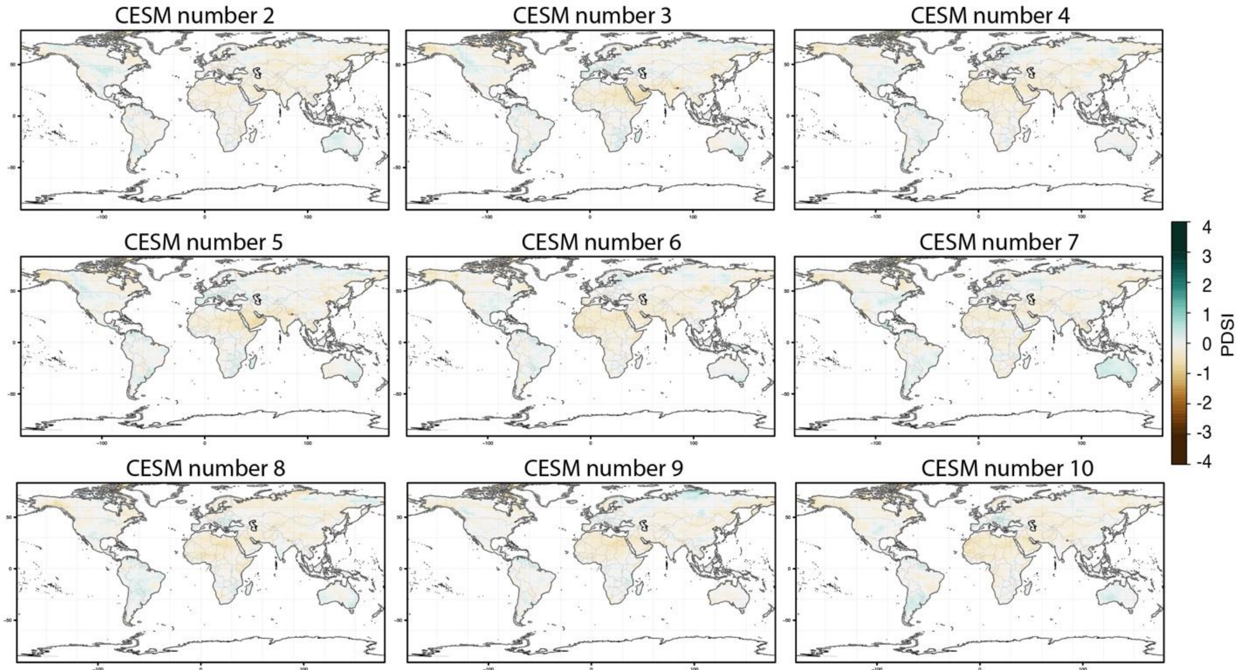

Fig. S10. Hydroclimatic response in 9 individual CESM ensemble members to TVE. Hydroclimatic response in 9 individual CESM ensemble members to Last Millennium large tropical volcanic events in the boreal winter. All maps show mean (years +0 to +20) PDSI after large tropical eruptions in DJF.

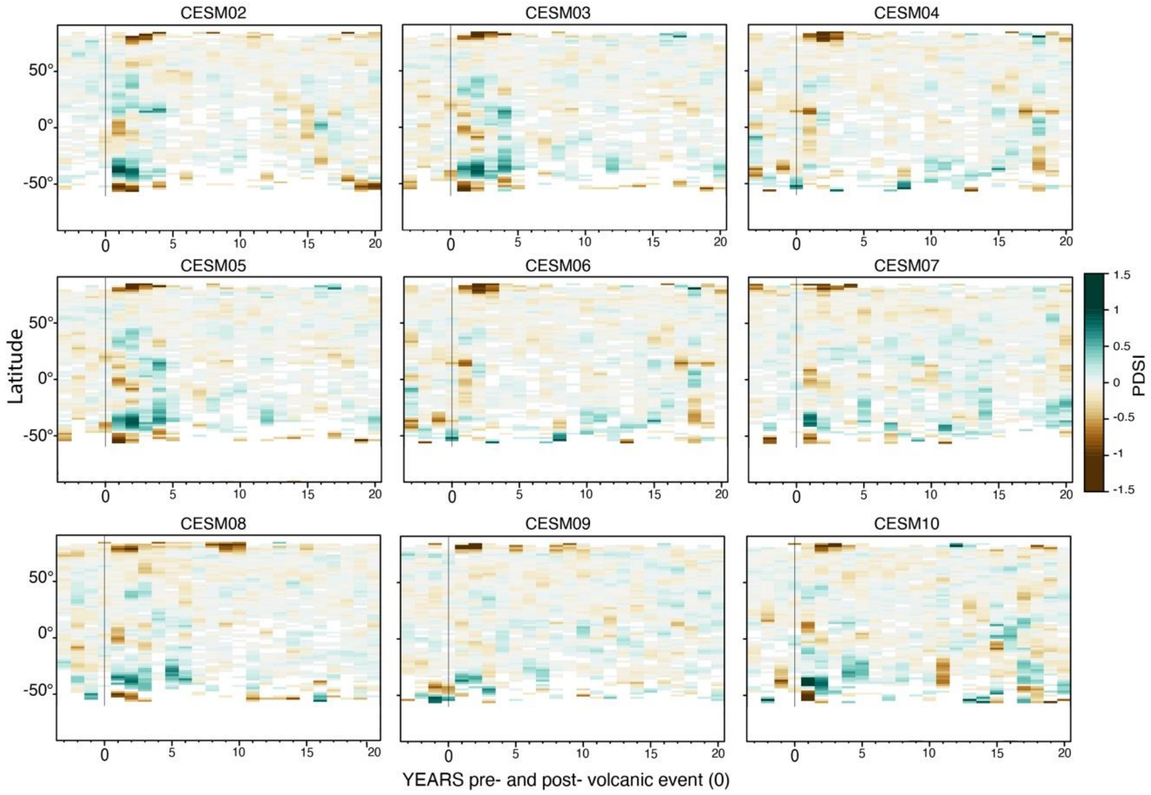

Fig. S11. Hovmöller diagram of the hydroclimatic response in 9 individual CESM ensemble members. Hovmöller diagram showing the weighted zonal-mean response to TVP in 9 individual CESM ensemble members to Last Millennium large tropical volcanic events in the boreal winter. Values that do not satisfy the significance threshold ($p<0.05$) are excluded. Vertical black line indicates the onset of the volcanic event. Greenland and Antarctica are not included.

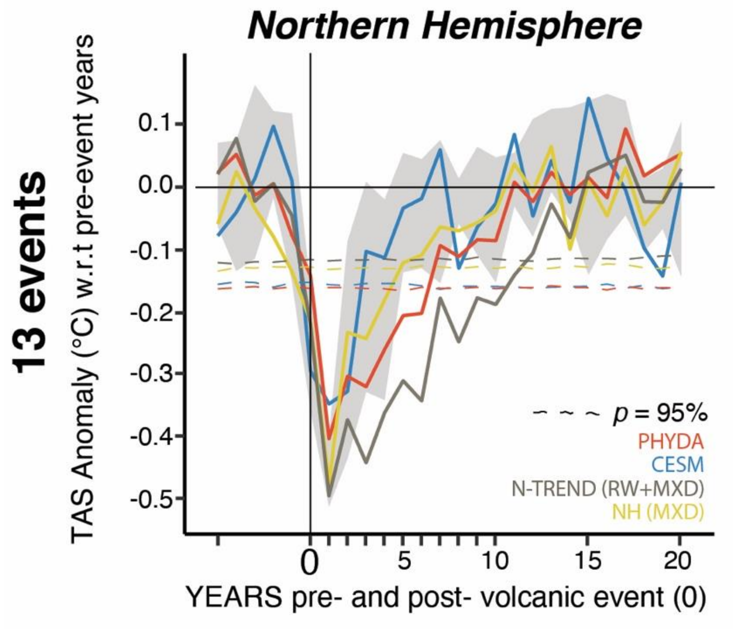

Fig. S12. Superposed Epoch Analysis (SEA) of the average northern hemisphere (> 25 ºN) temperature anomaly following 13 large tropical volcanic events (not including double-events). Events are taken over the last millennium estimated and use the JJA PHYDA, the JJA-CESM ensemble member number 10, and two proxy-based reconstructions (N-TREND and NH-MXD). The spread of the additional 9 CESM ensemble members is shown in grey. Additional reconstructions are N-TREND (*1*), which combines tree ring-width (TRW) with maximum latewood density measurements (MXD), and NH-MXD (*2*), based on MXD data only. Horizontal dashed lines represent significance at the 95% confidence-level for each estimate.

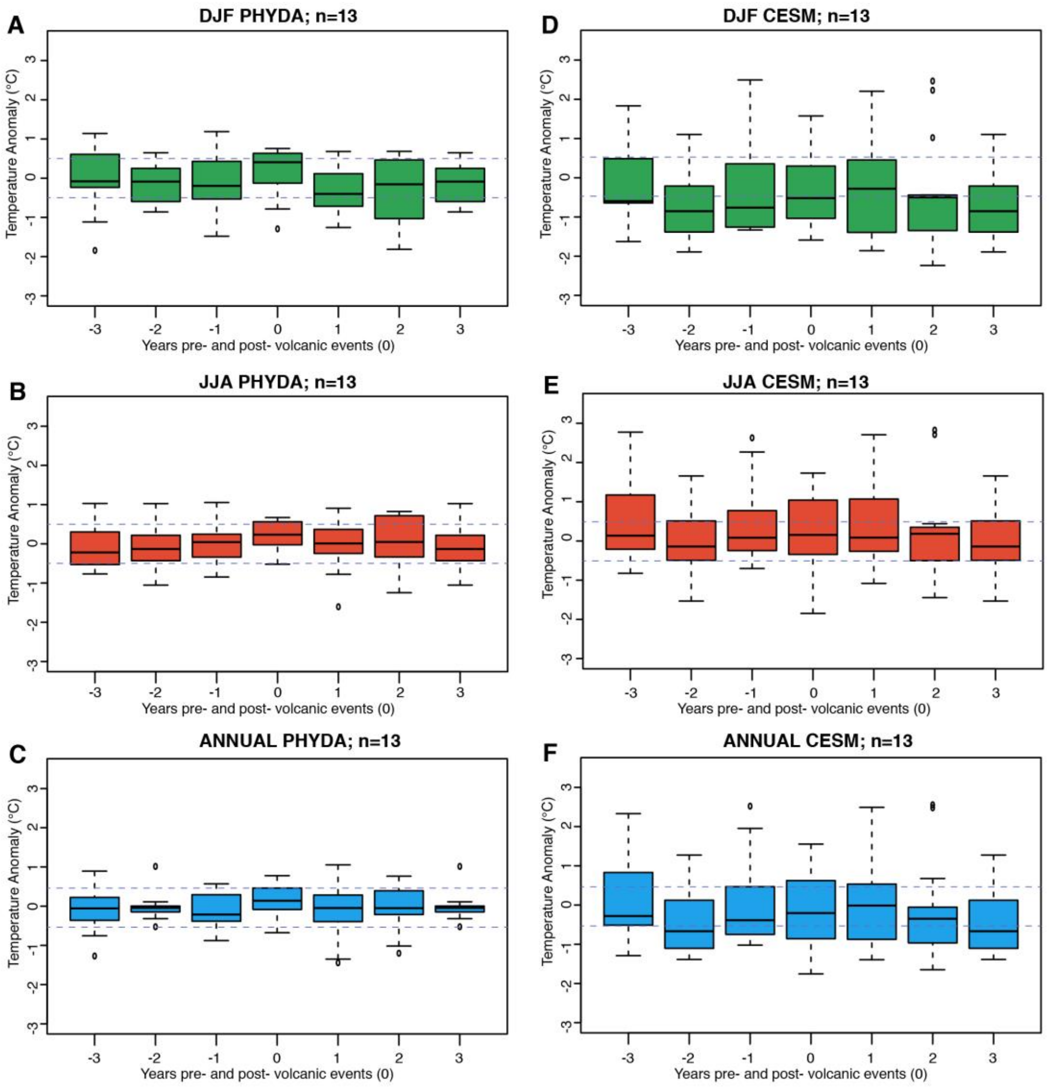

Fig. S13. ENSO response to TVEP and TVE. Boxplots showing the spread of the Niño3.4 state across the 13 events from 3 years prior to the volcanic event (0) to 4 years following the event in both seasons, as well as the annual average from PHYDA (left column) and CESM ensemble member 10 (right column). Dark center line in the boxplot represents the median, the edges of the boxes are 25th and 75th percentiles, and the whiskers extend to 1.5 times the median. Dashed lines represent the temperature anomaly thresholds indicating a more likely occurrence of El Niño (positive) or La Niña (negative) events.

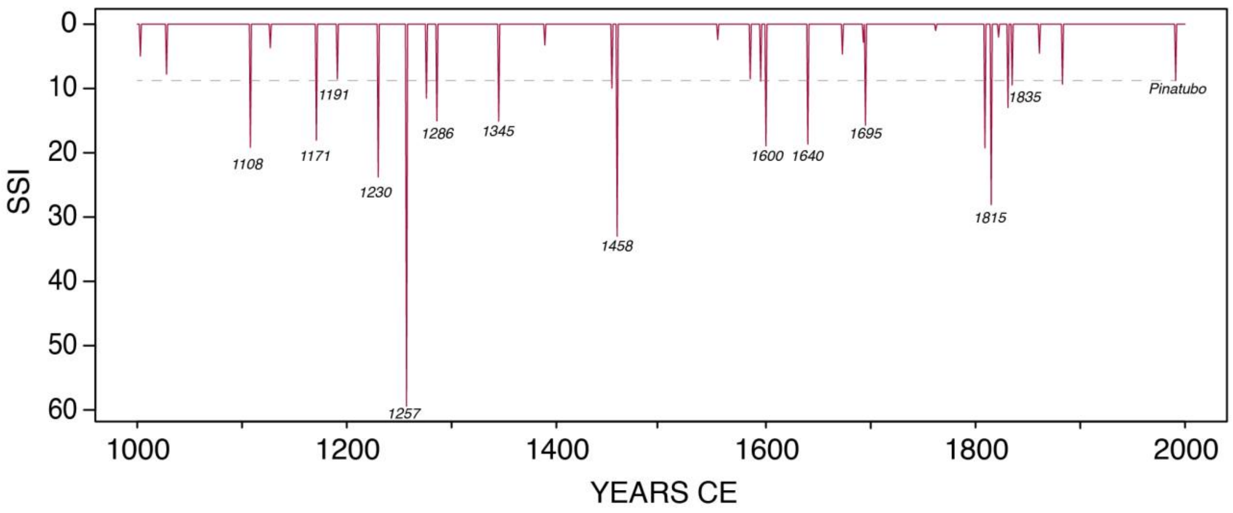

Fig. S14. Large millennium volcanic events larger than Pinatubo's 1991 magnitude. Last Millennium volcanic events larger than Pinatubo 1991 and associated stratospheric sulfur injection (SSI in Tg S). To avoid a potential bias introduced by 'double-events' (2 eruptions in short succession), only the events labeled with the year of the eruption were selected (n=13) for this study. Note that the Krakatoa eruption (1883) was not selected because CESM LME only spans 850 to 1850 CE.

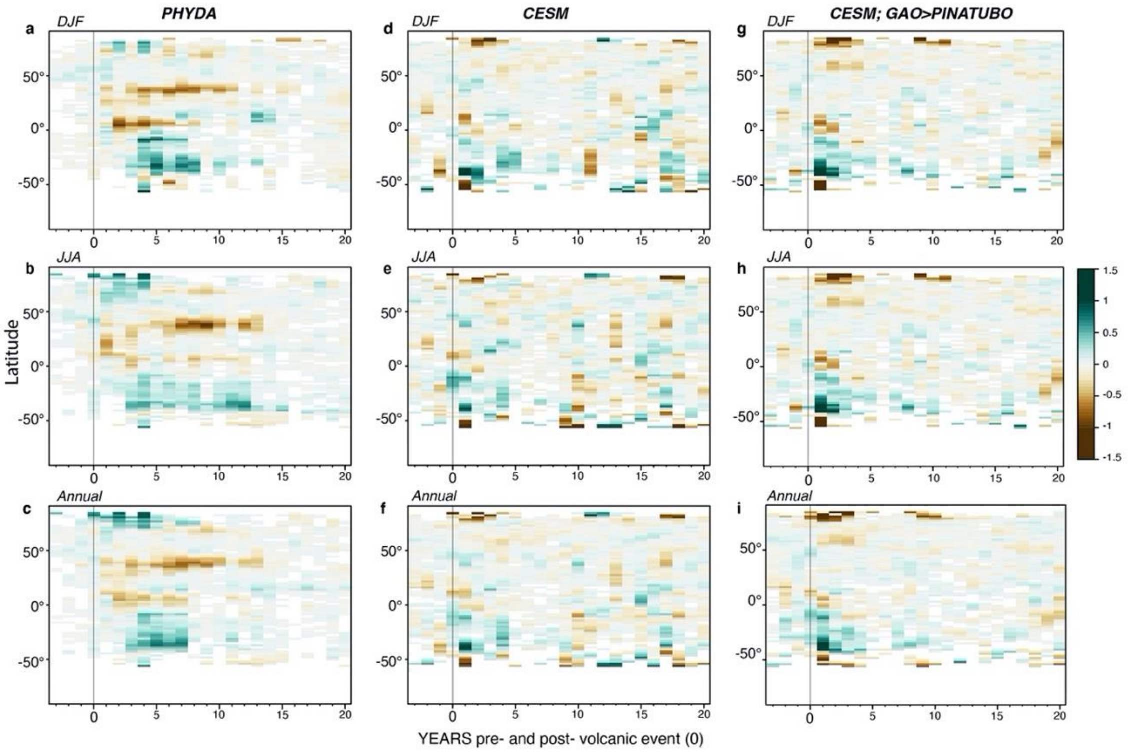

Fig. S15. **Hovmöller diagram showing the weighted zonal-mean hydroclimatic response in different seasons.** Results are derived from PHYDA (a, b, c) and eVolv2k events, CESM ensemble member 10 (d, e, f) and eVolv2k-adjacent events Ref. (*3*) volcanic reconstruction, and CESM ensemble member 10 using events greater than Pinatubo 1991 selected in the Ref. (*3*) volcanic reconstruction (g, h, i), respectively. Values that do not satisfy the significance threshold ($p<0.05$) are excluded. Vertical black line indicates the onset of the volcanic event. Greenland and Antarctica are not included.

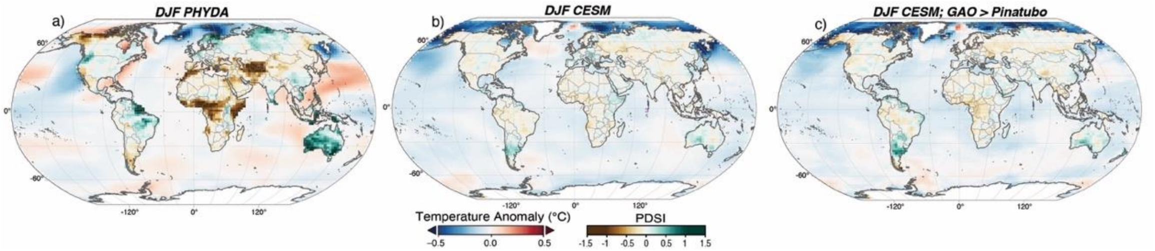

Fig. S16. Proxy and model estimates of the hydroclimatic and sea surface temperature (SST) responses to large tropical volcanic events (> the 1991 Mt. Pinatubo eruption) in the boreal winter. (a) SEA mean (years +0 to +20) of DJF SST and PDSI (land) using PHYDA and eVolv2k events; statistically insignificant values ($p>0.05$) are excluded in the mean. (b) As in (a) except from CESM ensemble member 10 (this ensemble member served as the PHYDA prior, but the characterized response is similar in all ensemble members) and eVolv2k-adjacent events in the Gao et al. (2008) volcanic reconstruction. (c) As in (b) except the SEA analysis was performed using volcanic events greater than Pinatubo 1991 (n=12) selected in the Gao et al. (2008) volcanic reconstruction.

Table S1. Selected climatic variables for this analysis. Selected climatic variables, seasons, and longitudinal coverage of variables used in this study and extracted or calculated from PHYDA and CESM. All the variables were considered for Annual, DJF, and JJA seasons. *Temperature is only used for the composite maps (Figure 3, and Figure S16), for the ocean (2m-marine air temperature, Figure S8), and for the Northern Hemisphere temperature SEA (Figure S12).

**Variable**	**Coverage**
Temperature*	Global (land, ocean)
PDSI	Global (land)
Niño 3.4	Lon. 120°W - 170°W
AMO	Lon. 25°W - 80°W
PDO	Lat. >20°N Pac. Ocean
ITCZ Tropical Africa	Lon. 15°W - 50°E
ITCZ Indian Ocean	Lon. 50°E - 95°E
ITCZ South Asia	Lon. 65°E - 95°E
ITCZ East Pacific	Lon. 130°E - 170°W
ITCZ Indonesia	Lon. 95°E - 130°E
ITCZ Atlantic Ocean	Lon. 40°W - 15°W
ITCZ South America	Lon. 100°W - 40°W
ITCZ West Pacific	Lon. 170°W - 100°W

Table S2. Selected volcanic events. List of the selected volcanic events according to each approach (Tropical volcanic events greater than Pinatubo 1991 (TVEP) with no overlap; or large Tropical Volcanic Events (TVE) with no overlap), including the Year CE and the volcanic stratospheric sulfur injection (VSSI) expressed in units of Tg sulfur.

**1. TVEP Year CE**	**VSSI**(*4*) **Tg [S]**	**2.TVE Year CE**	**VSSI**(*3*) **Tg [S]**
1108	19.1	*1110*	0.8
1171	18.0	*1167*	13.0
1191	8.5	*1195*	5.5
1230	23.8	*1227*	16.9
1257	59.4	*1258*	64.5
1286	15.0	*1284*	13.6
1345	15.1	*1341*	7.7
1458	32.9	*1459*	5.5
1600	18.9	*1600*	14.1
1640	18.6	*1641*	12.9
1695	15.7	*1693*	6.7
1815	28.0	*1815*	27.0
1835	9.4	*1835*	10.0

References

1. K. J. Anchukaitis, R. Wilson, K. R. Briffa, U. Büntgen, E. R. Cook, R. D'Arrigo, N. Davi, J. Esper, D. Frank, B. E. Gunnarson, G. Hegerl, S. Helama, S. Klesse, P. J. Krusic, H. W. Linderholm, V. Myglan, T. J. Osborn, P. Zhang, M. Rydval, L. Schneider, A. Schurer, G. Wiles, E. Zorita, Last millennium Northern Hemisphere summer temperatures from tree rings: Part II, spatially resolved reconstructions. *Quat. Sci. Rev.* **163**, 1–22 (2017).
2. L. Schneider, J. E. Smerdon, U. Büntgen, R. J. S. Wilson, V. S. Myglan, A. V Kirdyanov, J. Esper, Revising midlatitude summer temperatures back to A.D. 600 based on a wood density network. *Geophys. Res. Lett.* **42**, 4556–4562 (2015).
3. C. Gao, A. Robock, C. Ammann, Volcanic forcing of climate over the past 1500 years: An improved ice core-based index for climate models. *J. Geophys. Res.* **113**, D23111 (2008).
4. M. Toohey, M. Sigl, Volcanic stratospheric sulfur injections and aerosol optical depth from 500 BCE to 1900 CE. *Earth Syst. Sci. Data*. **9**, 809–831 (2017).
